# Second harmonic generation light quantifies the ratio of type III to total (I + III) collagen in a bundle of collagen fiber

**DOI:** 10.1038/s41598-021-91302-3

**Published:** 2021-06-04

**Authors:** Shukei Sugita, Takuya Suzumura, Akinobu Nakamura, Shinya Tsukiji, Yoshihiro Ujihara, Masanori Nakamura

**Affiliations:** 1 Department of Electrical and Mechanical Engineering, Nagoya Institute of Technology, Gokiso-cho, Showa-ku, Nagoya, 466-8555 Japan; 2 Center of Biomedical Physics and Information Technology, Nagoya Institute of Technology, Nagoya, Japan; 3 Department of Life Science and Applied Chemistry, Nagoya Institute of Technology, Nagoya, Japan; 4 Department of Nanopharmaceutical Sciences, Nagoya Institute of Technology, Nagoya, Japan

**Keywords:** Biological techniques, Microscopy, Laboratory techniques and procedures

## Abstract

The ratio of type III to type I collagen is important for properly maintaining functions of organs and cells. We propose a method to quantify the ratio of type III to total (type I + III) collagen (*λ*_*III*_) in a given collagen fiber bundle using second harmonic generation (SHG) light. First, the relationship between SHG light intensity and the *λ*_*III*_ of collagen gels was examined, and the slope (*k*_1_) and SHG light intensity at 0% type III collagen (*k*_2_) were determined. Second, the SHG light intensity of a 100% type I collagen fiber bundle and its diameter (*D*) were measured, and the slope (*k*_3_) of the relationship was determined. The *λ*_*III*_ in a collagen fiber bundle was estimated from these constants (*k*_1-3_) and SHG light intensity. We applied this method to collagen fiber bundles isolated from the media and adventitia of porcine thoracic aortas, and obtained *λ*_*III*_ = 84.7% ± 13.8% and *λ*_*III*_ = 17.5% ± 15.2%, respectively. These values concurred with those obtained with a typical quantification method using sodium dodecyl sulfate–polyacrylamide gel electrophoresis. The findings demonstrated that the method proposed is useful to quantify the ratio of type III to total collagen in a collagen fiber bundle.

## Introduction

Collagen is the most common protein in the human body^1^, with useful mechanical properties such as high rigidity (elastic modulus 1 GPa) and strength (tensile strength 50–100 MPa)^2^, thereby providing structural stability and strength to various tissues and organs. To date, more than 28 subtypes of collagen have been identified in the human body^3^; these subtypes comprise a combination of three α chains and several amino acids. Among these, type I and III collagen are the most common ones, accounting for 70% and 5–20%, respectively, of total collagen in mammals^4^. Furthermore, type I and III collagen account for 80–85% and 10–15%, respectively, of total collagen in human skin^5^ and 85% and 11%, respectively, of total collagen in rat hearts^6^.

Type I/III collagen ratio is important for maintaining the functions and mechanical properties of various organs and cells. An increase in the type I/III collagen ratio is associated with a decrease in cardiac output and dysfunction such as dilated cardiomyopathy^7–9^. Conversely, a decrease in the type I/III collagen ratio has been associated with a decrease in the severity of ischemic cardiomyopathy in rat models by improving cardiac contractile function and left ventricular remodeling^10^. An optimum ratio of type I/III collagen is also required to maintain normal bladder tension and contraction^11^. Type I/III collagen ratio is an indicator of skin wound healing^12, 13^. Alterations in type III procollagen are associated with malignant transformations in ovarian tumors^14^ as well as with the proliferation and metastatic potential of breast cancer cells^15^. These research findings indicate that quantification of type III collagen can be used as a diagnostic tool for various diseases.

Currently, there are several methods to investigate the type I/III collagen ratio such as those involving sodium dodecyl sulfate–polyacrylamide gel electrophoresis (SDS–PAGE), tissue staining, and gene expression analysis. However, these methods are time-consuming and require at least several hours to obtain results. SDS–PAGE and gene expression analysis provide only bulk properties of samples, whereas tissue staining is limited in terms of quantification accuracy. Therefore, further research is necessary to develop a suitable method to determine the ratio of type I/III collagen locally and accurately in a short time.

Against this background, this study has presented a novel method to quantify the ratio of type III to total (type I + type III) collagen in a bundle of collagen fiber using second harmonic generation (SHG) light. The method was applied to bundles of collagen fiber isolated from the media and adventitia of porcine thoracic aortas. We verified the method’s accuracy by comparing the ratio of type III collagen/total collagen estimated using our proposed method with that obtained using SDS–PAGE.

## Strategy

For this study, we assumed that the collagen fiber was made up of type I and type III collagen only, a common assumption adopted in similar studies^4–6^. Hereafter, *λ*_*III*_ refers to the type III collagen/total collagen ratio, calculated from the equation below:

1 $$\lambda_{III} = \frac{{V_{III} }}{{V_{I} + V_{III} }}$$

where *V*_*I*_ and *V*_*III*_ are the volumes of type I and III collagen, respectively, in a sample.

**Figure 1:**
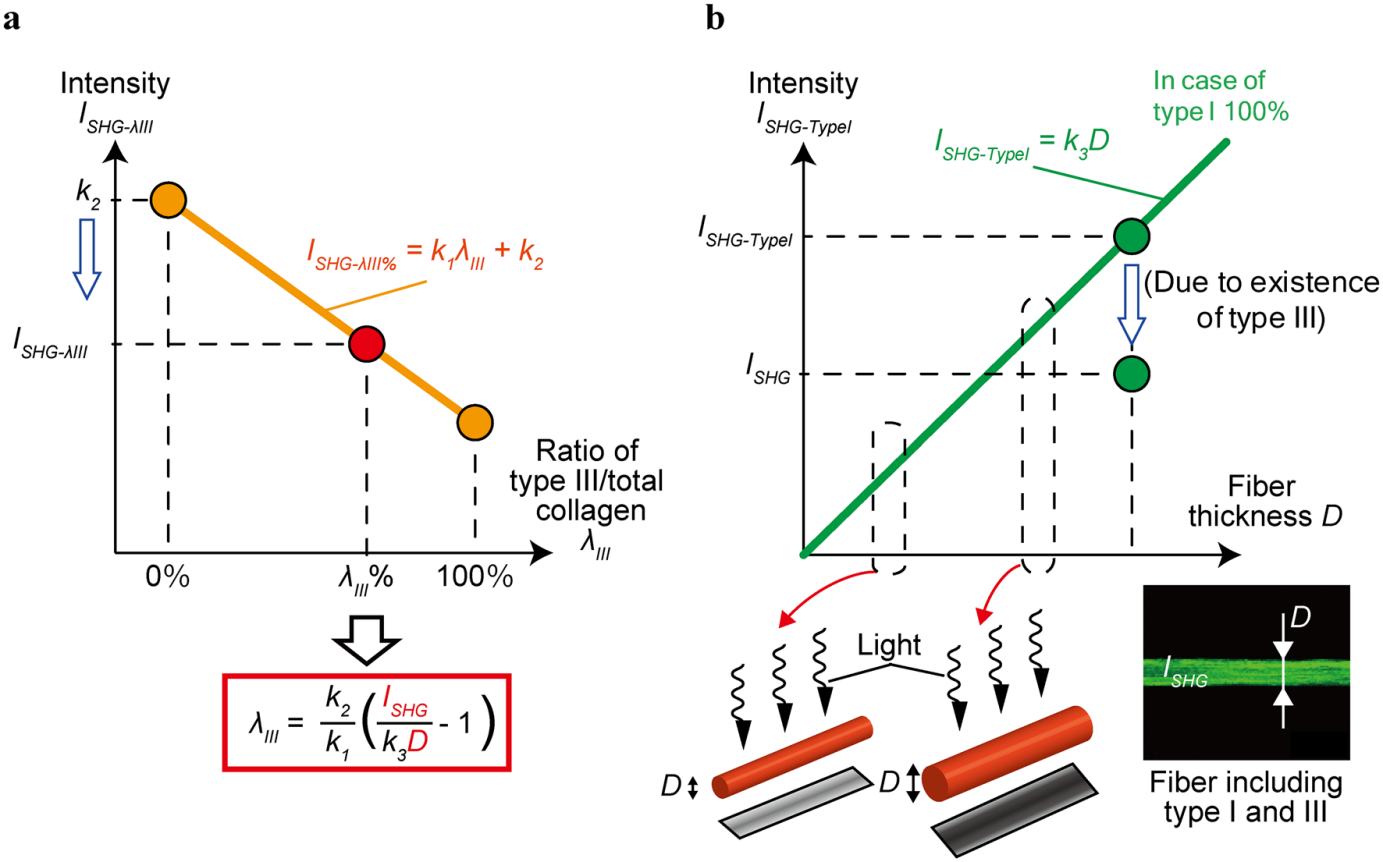
Two equations about SHG intensities are required to estimate *λ*_*III*_. Two relationships of type III ratio–SHG intensity (*λ*_*III*_ – *I*_*SHG-λIII*_) (**a**) and fiber bundle diameter–SHG intensity of type I collagen (*D*–*I*_*SHG-TypeI*_) (**b**) were obtained from separately performed experiments to determine *k*_1_, *k*_2_, and *k*_3_. For a tested collagen fiber bundle where *λ*_*III*_ is unknown, *D* and SHG light intensity (*I*_*SHG*_) of the fiber bundle were measured (picture in the right bottom in (**b**). *I*_*SHG-TypeI*_ was calculated from the *D* in the graph (**b**). Since the ratio of *I*_*SHG*_:*I*_*SHG-TypeI*_ equals to the *I*_*SHG-λIII*_:*k*_2_, *λ*_*III*_ can be determined. This study used the diameter of fiber bundle because the diameter should be proportional to the amount of collagen fiber bundle. This figure was created using Adobe Illustrator (Adobe, CS6).

A schematic of the strategy to estimate the ratio of type III to total collagen, reflected by *λ*_*III*_, in a bundle of collagen fiber is shown in Fig. 1. SHG light intensity is a function of *λ*_*III*_ and the volume of a bundle of collagen fiber. We use these two functions to estimate *λ*_*III*_ in each collagen fiber bundle.

The first relationship is that SHG light intensity (*I*_*SHG-λIII*_) decreases linearly with an increasing *λ*_*III*_ (Fig. 1a), which was described by Lutz et al.^16^. Mathematically, this relationship is expressed as follows:

2 $$I_{SHG - \lambda III} = k_{1} \lambda_{III} + k_{2}$$

where *k*_1_ and *k*_2_ are constants to be determined from the experiments. Constant *k*_1_ means the slope of the fitted line and *k*_2_ is the SHG light intensity at *λ*_*III*_ = 0 (*i.e.*, when the sample is made of pure type I collagen). Thus, the ratio of SHG light intensities (*δ*) of samples including type III collagen to pure type I collagen samples is expressed by:

3 $$\delta = \frac{{I_{SHG - \lambda III} }}{{I_{SHG - TypeI - gel} }} = \frac{{I_{SHG - \lambda III} }}{{k_{2} }},$$

where *I*_*SHG-TypeI-gel*_ is SHG intensity of samples constituted from pure type I collagen.

The second important relationship highlighted by Lutz et al.^16^ is that the SHG light intensity in pure type I collagen (*I*_*SHG-TypeI*_) increases linearly with an increasing total collagen volume (Fig. 1b). In our study, collagen fiber diameter (*D*) was used as a substitute to reflect total collagen volume (Fig. 1b). The relationship between SHG light intensity and collagen diameter was calculated as follows:

4 $$I_{SHG - TypeI} = k_{3} D$$

where *k*_3_ is a constant expressing the slope of the Eq. (4).

When attempting to estimate *λ*_*III*_ of a given sample of collagen fiber bundle with a diameter *D*, we first compare the measured SHG light intensity of the actual collagen fiber bundle *I*_*SHG*_ with that predicted if the bundle was made up of pure type I collagen (*I*_*SHG-TypeI*_), calculated from Eq. (4). The *I*_*SHG*_ value should be lower than the *I*_*SHG-TypeI*_ value because the bundle comprises type III collagen. From this difference, we can estimate the ratio of SHG light intensity *δ* (Eq. 3) caused by collagen type III fiber, expressed as follows:

5 $$\delta = \frac{{I_{SHG} }}{{I_{SHG - TypeI} }}.$$

Hence, to estimate the *λ*_*III*_ of a given collagen fiber bundle, we can combine Eqs. (2)–(5) to obtain the following equation:

6 $$\lambda_{III} = \frac{{k_{2} }}{{k_{1} }}\left( {\frac{{I_{SHG} }}{{k_{3} D}} - 1} \right).$$

Equation (6) tells that the *λ*_*III*_ of the collagen fiber bundle can be estimated from the diameter *D* and SHG light intensity *I*_*SHG*_ if the constants *k*_1_, *k*_2_, and *k*_3_ are readily determined.

In this study, to determine the relationship between fiber bundle diameter and SHG light intensity for type I collagen (*D*–*I*_*SHG-TypeI*_), tail tendon samples from rats were used owing to the high concentration of type I collagen (95%) in these samples^17^. This relationship (*D*–*I*_*SHG-Tendon*_) can be described as follows:

7 $$I_{SHG - Tendon} = k_{3}^{\prime } D$$

where *k*_3_*′* is a constant determined from experiments and denotes the slope of the Eq. (7). Type III/total collagen ratio, reflected by *λ*_*III*_, of the tendon is estimated as *λ*_*III-Tendon*_. The ratio of SHG light intensities in the tendon (*δ*_*Tendon*_) including type III collagen to pure type I collagen can be determined by combining Eqs. (2) and (3) as follows:

8 $$\delta_{Tendon} = \frac{{k_{1} \lambda_{III - Tendon} + k_{2} }}{{k_{2} }},$$

where *I*_*SHG-TypeI*_ is calculated as follows (adapted from Eq. 4):

9 $$\begin{aligned} I_{SHG - TypeI} = & \frac{{I_{SHG - Tendon} }}{{\delta_{Tendon} }} \\ = & \frac{{k_{2} k_{3}^{\prime } D}}{{k_{1} \lambda_{III - Tendon} + k_{2} }} \\ = & k_{3} D. \\ \end{aligned}$$

Equation (9) shows that the constant *k*_3_ is determined from the constants *k*_1_, *k*_2_, *k*_3_*ʹ*, and *λ*_*III*_ in the tendon fiber *λ*_*III-Tendon*_, which are determined experimentally.

## Results

### Relationship between SHG light intensity and type III/total collagen ratio

**Figure 2:**
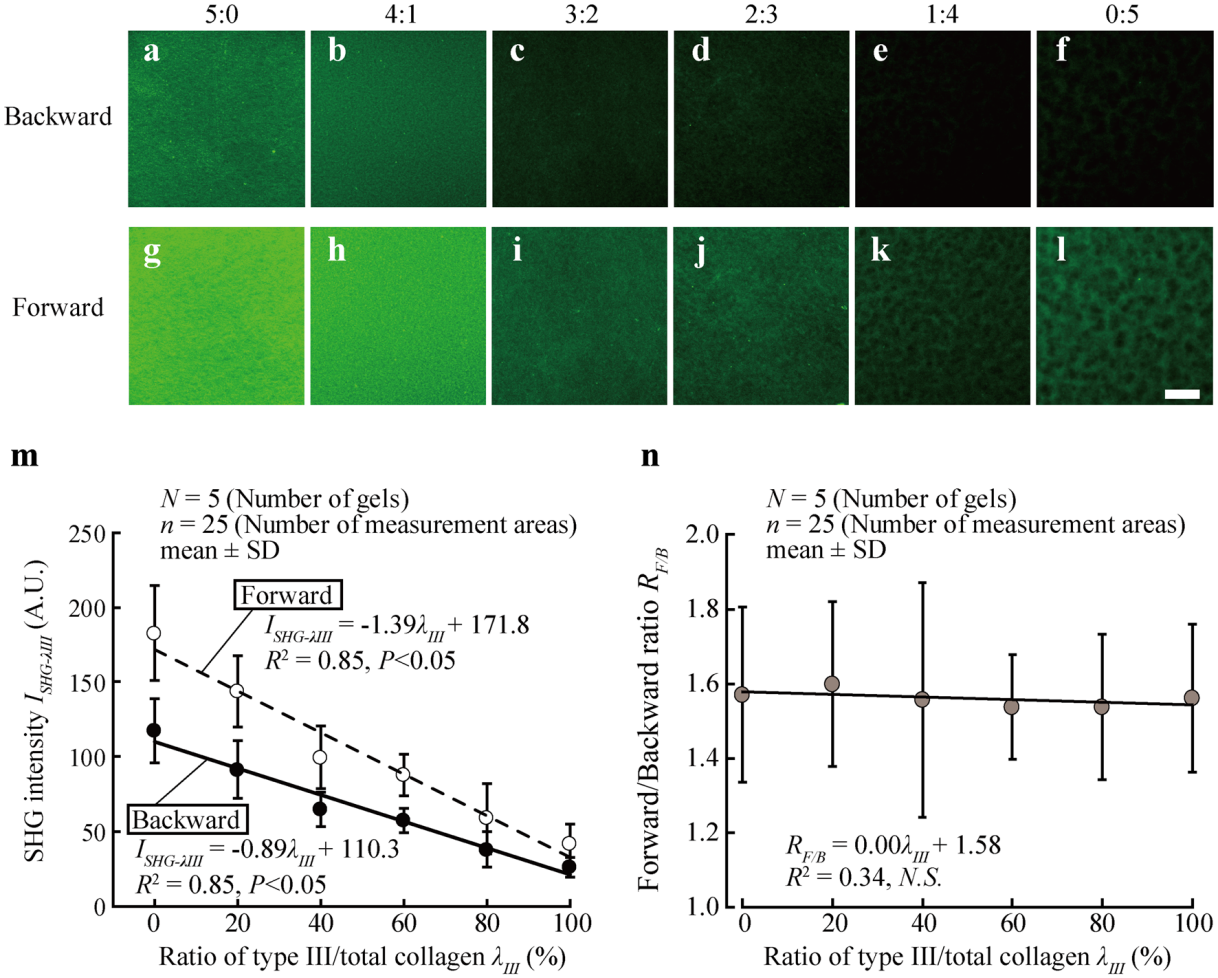
SHG intensity *I*_*SHG-λIII*_ linearly decreases with an elevation of *λ*_*III*_. (**a**–**l**) Typical SHG images of collagen gels captured in backward (**a**–**f**) and forward (**g**–**l**) photomultiplier tubes. The *λ*_*III*_ was changed for each gel. Scale bar = 100 µm for all images. (**m**) Plot showing the relationship between SHG light intensity and *λ*_*III*_. (**n**) Forward/backward ratio of SHG light intensity plotted against *λ*_*III*_.

The relationship between *I*_*SHG-λIII*_ and *λ*_*III*_ was determined using collagen gels made by mixing type I and III collagen. Figure 2a–l shows typical SHG light images of gels mixed with type I and III collagen. The SHG intensity *I*_*SHG-λIII*_ of collagen gel appears to decrease with *λ*_*III*_ increase in both the backward and forward directions of photomultiplier tubes. In the backward direction, SHG intensity was lower than in the forward direction. Our quantitative analysis confirmed these results (Fig. 2m). The SHG intensity *I*_*SHG*_ of the collagen gel was significantly correlated with *λ*_*III*_ in both the backward and forward directions (*R*^2^ = 0.85, *P* < 0.001). From this estimation, we were able to determine constants *k*_1_ =  − 1.39 and *k*_2_ = 171.8 in the forward and *k*_1_ =  − 0.89 and *k*_2_ = 110.3 in the backward directions as described in Eq. (2). In further analyses, the SHG signal was collected in the backward direction to minimize the effects of fiber orientation and organization on the image^18^. Figure 2n shows the forward/backward ratio of SHG light intensity, which was almost constant in all samples examined, with no significant correlation observed (*R*^2^ = 0.34, *P* = 0.98). This is in contrast to a previous study^19^, which reported that the forward–backward ratio of SHG light intensity decreased with an increase in type III collagen concentration.

### Relationship between SHG light intensity and diameter of fiber bundles for type I collagen

**Figure 3:**
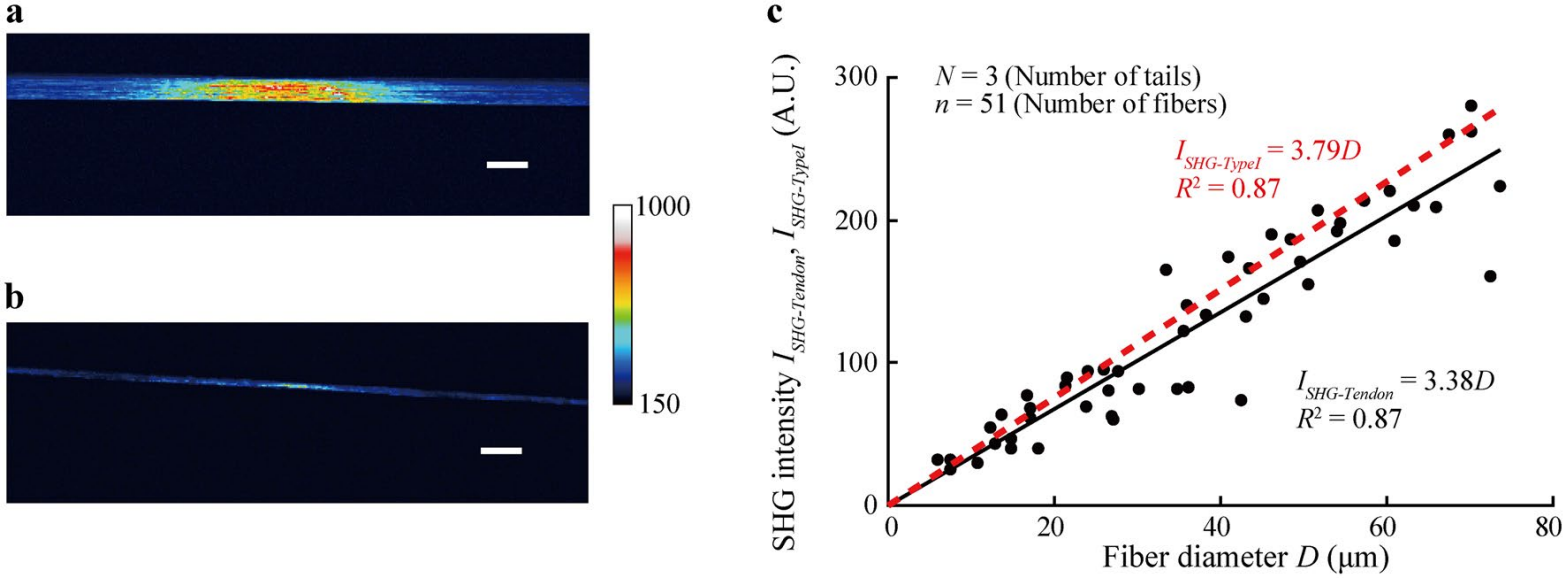
SHG light intensity *I*_*SHG*_ increases with increase in the diameter *D* of collagen fiber bundle. (**a**, **b**) Typical images of SHG light of (**a**) thick (*D* = 67.5 µm) and (**b**) thin (*D* = 17.0 µm) collagen fiber bundles obtained from rat tail tendons. Scale bars = 100 µm. (**c**) Relationship between SHG light intensity *I*_*SHG-Tendon*_ and the diameter *D* of collagen fiber bundles. Raw data plots were fitted (black straight line) with a least-squares regression. The red dashed line represents the hypothetical relationship when fibers were assumed to be composed of 100% type I collagen (*I*_*SHG-TypeI*_), obtained by compensation using *λ*_*III*_*-*_*Tendon*_.

Constant *k*_3_*′* in the relationship between *D* and *I*_*SHG-TypeI*_ of a type I collagen fiber bundle was determined in rat tail tendons. Figure 3a,b show typical images of collagen fiber bundles obtained from the rat tail tendons in this study. The bundle diameter appears to be constant for each sample. SHG light intensity *I*_*SHG-Tendon*_ is significantly correlated with the bundle diameter (*R*^2^ = 0.87, *P* < 0.05; Fig. 3c). From this relationship, we determined *k*_3_^ʹ^ = 3.38 in Eq. (7).

### ***λ***_***III***_ of collagen fibers measured with SDS–PAGE

SDS–PAGE was conducted for two different samples. First, SDS-PAGE was applied to collagen fiber bundles of rat tail tendons to determine *k*_3_ in Eq. (9) by obtaining *λ*_*III-Tendon*_*.* Second, SDS-PAGE was applied to collagen fiber bundles of porcine aorta to check the validity of the proposed method.

**Figure 4:**
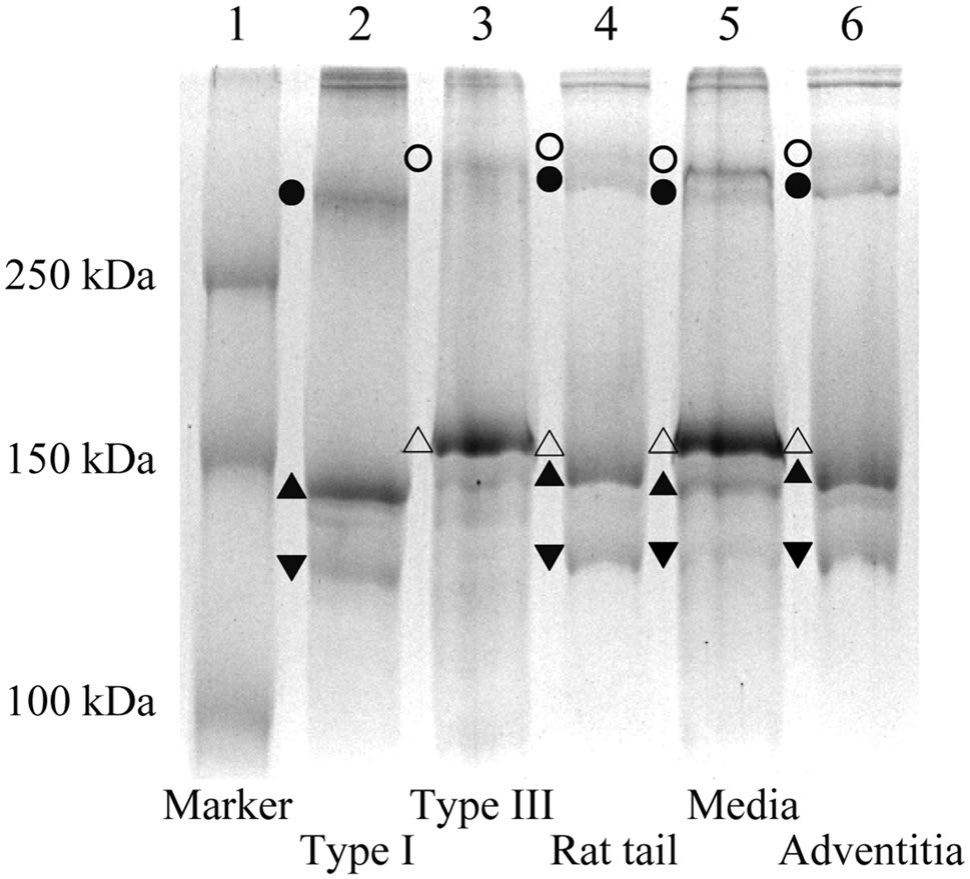
SDS–PAGE including urea differentiates type I and III collagen. Scanned image of a gel obtained using SDS–PAGE. Markers show the collagen bands of *α*_1_(I) (filled triangle), *α*_2_(I) (filled inverted triangle), *β*(I) (filled circle), *α*_1_(III) (triangle), and *β*(III) (circle).

Figure 4 shows a typical image obtained using SDS–PAGE. Although the molecular weight of type I collagen (283.3 ± 1.3 kDa) is similar to that of type III (288.3 ± 1.3 kDa)^20^, including urea in this method successfully distinguished these bands (lanes 2 and 3 in Fig. 4). Collagen fiber bundles from rat tail tendon (lane 4) and adventitia (lane 6) have a higher density of type I collagen (α_1_(I), α_2_(I), and β(I)) compared to type III collagen (α_1_(III) and β(III)), while collagen from media (lane 5) shows a lower density of type I collagen compared to type III collagen.

According to SDS–PAGE, the *λ*_*III*_ in rat tail tendons (*λ*_*III-Tendon*_) was 13.1% ± 3.8%, lane 4 in Fig. 4). Thus, we calculated the gradient of the relationship between SHG light intensity *I*_*SHG-TypeI*_ and diameter *D* in a hypothetical 100% type I collagen fiber bundle, then determined constant *k*_3_ = 3.79 as shown in Eq. (4) (Fig. 3c). Our quantitative analysis showed that the *λ*_*III*_ of the aortic media obtained with SDS–PAGE was 73.9% ± 1.5% and that of the adventitia was 15.5% ± 5.1%.

### ***λ***_***III***_ of collagen fiber bundles of thoracic aorta using SHG light intensity

**Figure 5:**
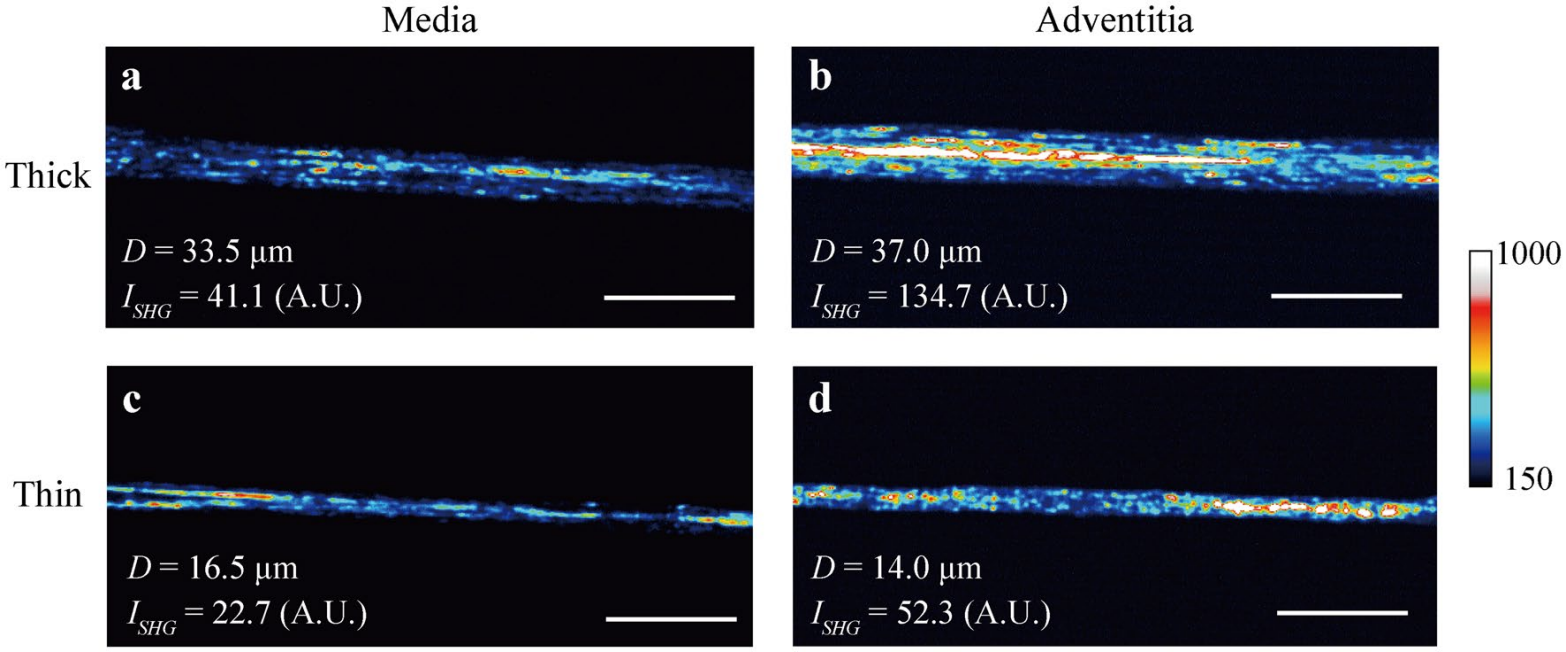
SHG light intensity *I*_*SHG*_ increases in adventitial and thick fiber bundles than medial and thin fiber bundles. Typical images of SHG light of (**a**, **b**) thick and (**c**, **d**) thin fiber bundles obtained from (**a**, **c**) media and (**b**, **d**) adventitia of porcine thoracic aorta. Scale bars = 100 µm.

The method proposed was applied to collagen fiber bundles isolated from the media and adventitia of porcine thoracic aortas. Figure 5 shows typical images of collagen fiber bundles obtained from the media and the adventitia of porcine thoracic aortas with 5 × optical zoom (see Supplementary Information S1 online, image without optical zoom). When comparing bundles of similar diameters, samples obtained from the media (Fig. 5a) showed a lower SHG intensity than those obtained from the adventitia (Fig. 5b). This tendency was also observed in thin bundles (Fig. 5c,d); in both the media and the adventitia, thick bundles (Fig. 5a,b) had higher intensities than thin bundles (Fig. 5c,d).

**Figure 6:**
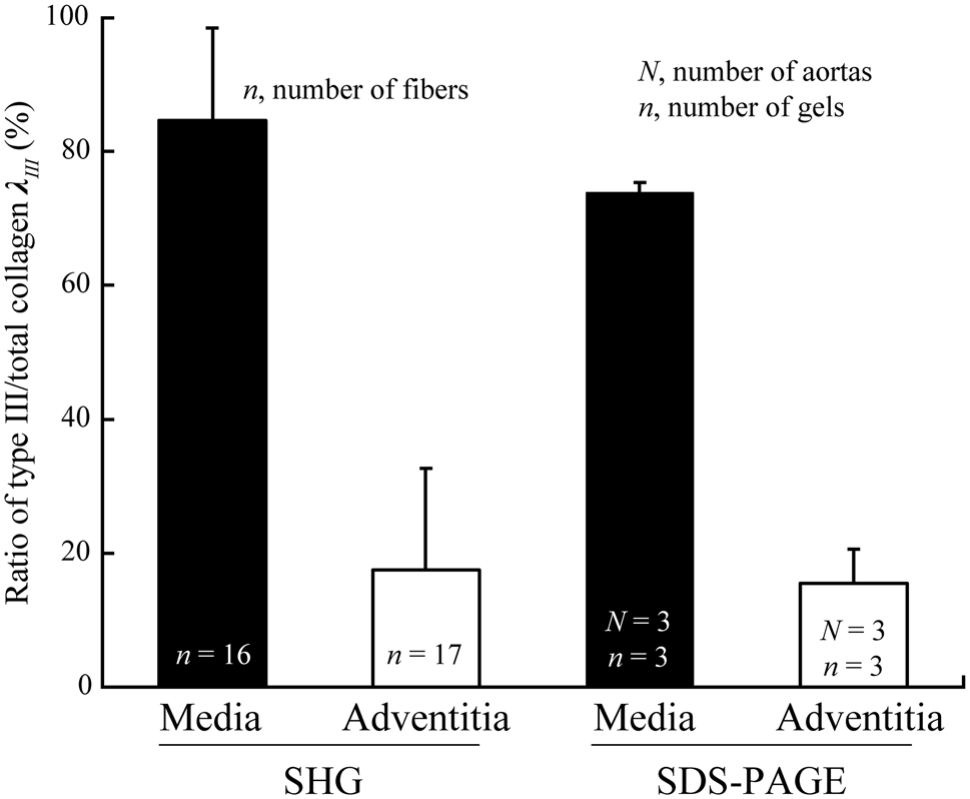
*λ*_*III*_ estimation by the proposed method concurred the data obtained using SDA-PAGE. Quantified *λ*_*III*_ of medial and adventitial collagen fiber bundles in porcine thoracic aortas using the SHG method suggested in this study and SDS–PAGE. Data are shown as means + SD.

The *λ*_*III*_ of the medial and the adventitial collagen fiber bundles were calculated using Eq. (6). Figure 6 shows the *λ*_*III*_ value obtained from the SHG method along with that of SDS–PAGE. The *λ*_*III*_ was significantly higher in the medial collagen (84.7% ± 13.8%, *n* = 16) than in the adventitial (17.5% ± 15.2%, *n* = 17). These results were similar to those obtained using SDS–PAGE.

## Discussion

In this study, we proposed a new method to quantify the ratio of type III to total collagen (*λ*_*III*_) of a given fiber bundle from its diameter and SHG light intensity. We also confirmed the validity of our method against a known procedure (SDS–PAGE). Our method is based on two relationships: the first between type I collagen diameter and SHG light intensity; the second between *λ*_*III*_ and SHG light intensity^16^. Combining these enables us to quantify *λ*_*III*_ within a collagen fiber bundle. As the measurement of *λ*_*III*_ using previous methods is time-consuming (taking at least several hours), our method is beneficial. We applied this method to collagen fiber bundles obtained from the media and the adventitia of porcine thoracic aortas and found that *λ*_*III*_ in the media and adventitia concurred with the data obtained using SDS–PAGE (Fig. 6). Moreover, these results also concur with other previous studies: the ratio of type III and I to total collagen in the human aortic media are 70% and 30%, respectively^21^ and adventitia is almost exclusively composed of type I collagen^22^. Thus, we can say that our proposed method is suitable for the evaluation of *λ*_*III*_.

An increase in the ratio of type III resulted in a decrease in the SHG intensity (Fig. 2). Tilbury^19^ reported that relative increase in the type III ratio within fibrils induced changes in the structure of collagen fibrils: decrease in fibrillary organization shown by shorter and more randomly arranged collagen fibers: decrease in the dipole moment shown by SHG signal anisotropy within fibers: an increase in the pitch angle of α-helices shown by pixel-based polarization-resolved approach. Such changes in the structure of fibrils with increasing type III collagen would have decreased SHG intensity with an increase in the type III ratio.

Our SHG light images contained heterogeneous intensity distributions within the collagen fiber bundles observed. The intensity at the center of the fiber bundle image was higher than in the peripheral area (Fig. 3a,b). This appears to be caused by the higher laser power in the central region of the images by the optical devices used in this study. To avoid this heterogeneity, we used the same setting throughout the experiment. However, heterogeneous distribution can be seen even within a collagen fiber bundle on a scale of only a few micrometers (e.g., in Fig. 5). It is not clear whether the heterogeneous distribution reflects the actual spatial difference in the local density of type III collagen, as there is no method to verify this hypothesis. Since the level of the standard deviation in SHG intensity shown in Fig. 5 is much larger than that of Fig. 2, this high frequency is not caused by noise. Previous studies demonstrated that glycation of collagen decreased SHG intensity^23^ and a lower level of the crosslinking increased SHG intensity^24^. It is thus considered that the heterogeneity of SHG light intensity is possibly produced by the local non-enzymatic glycation and lysyl oxidase-mediated crosslinking of collagen.

Our proposed method has several advantages: first, it allows researchers to quantify *λ*_*III*_ more quickly than existing methods such as SDS–PAGE, gene expression^25^, and immunofluorescence^26^, and picrosirius red staining^27^. Our method only requires a collagen fiber bundle to be placed and observed under a microscope, which is much quicker and handier than previous methods. Potential applications of the proposed method include cosmetic, healthcare, and clinical research. Second, this method measures *λ*_*III*_ without the need for invasive procedures such as fixation and homogenization. Therefore, we can simultaneously measure multiple properties (*e.g.*, mechanical properties) from a fiber bundle.

Although the method proposed in this study has not been fully established, it has wide potential for practical application. For instance, this method can be applied to tissues in vivo as it uses SHG light in the backward direction (*i.e.*, the light does not have to penetrate through the whole thickness of the specimen). Since the SHG light of human skin in vivo can be observed^28, 29^, *λ*_*III*_ of the skin can be estimated. Although our method requires the isolation of a collagen fiber bundle from a given tissue, techniques to quantify the volume of collagen in tissues enable us to measure the *λ*_*III*_ of skin, albeit invasively. Moreover, the spatial distribution of *λ*_*III*_ in a specimen can be obtained. Although we have to investigate whether the distribution of intensity is accurate, measuring the local distribution of type I/III ratio in tissues can be used as an indicator for skin wound healing^12, 13^.

Users have to bear in mind that this method uses the intensity. When the SHG light of a collagen fiber bundle is repeatedly captured, the intensity is slightly decreased after every image captured (see Supplementary Information S2 online). Although this decrease was small (0.4% after each image capture) and did not affect the results of this study, this decay might affect results in future measurements and should thus be taken into account. Furthermore, the light intensity varies depending on the devices used, such as lasers, quantum efficiency and sensitivity of photon multiplier tubes, as well as optical devices (*e.g.*, objectives and filters). To use our proposed method, users will need to adjust the constant parameters *k*_1_, *k*_2_, and *k*_3_ in their system. Preparing samples of the various concentrations of type III collagen and several diameters of collagen fiber bundles can help establish a reference standard.

In this study, we assumed that the collagen fiber was made up of type I and type III collagen only, as commonly assumed in past studies^4–6^. Reportedly, collagen compositions of human arteries were type I of 60%, III of 30% and others (type V and minor collagens) of 10%^30^, indicating that type I and III were dominant compared to other types. These compositions are obtained from human tissues, not from the porcine ones used in this study. Carrasco et al^31^ implemented comparative studies on the collagen of vertebrate arteries, and reported that vertebrates shared morphologic and histochemical features of collagens in arteries. These reports suggest that it could be reasonable to assume that the porcine aorta is made mainly of type I and type III collagens like the human aorta, although they are not identical.

Care for other types of collagen and non-collagenous tissues is needed in the application of the proposed method to actual biological tissues. The potential effects of non-collagenous proteins, for example, collagen-bound small leucine-rich proteoglycans that regulate collagen fibril formation and possibly intermolecular space, on SHG cannot be entirely excluded. SHG signals thus may include signal generated from non-collagenous proteins. Type II collagen is the major component in articular cartilages^32^. Because type II collagen generates strong SHG light^33^, direct application of the proposed method to such tissues might not be adequate. Type IV and type V collagen are relatively common in tissues, but they do not generate the SHG light^33^. Elastin and cells also do not affect SHG light, because SHG light generates from noncentrosymmetric molecular assemblies^34^. In support of it, high SHG light intensity areas do not colocalize with non-collagenous tissue regions^18, 32^. These indicate that the method can be applied to most biological tissues except type II collagen-rich ones such as articular cartilage.

In SDS–PAGE, *λ*_*III*_ was quantified with the β-component and α chains to obtain a more accurate measurement. To consider the effect of the β-component on our result, we also measured the *λ*_*III*_ using only the bands of α chains. The values obtained from the rat tail tendon and the aortic media and adventitia were 10.9% ± 3.0%, 75.3% ± 2.5%, and 11.6% ± 3.5%, respectively. Compared with the results shown in Fig. 6, the *λ*_*III*_ value showed a < 3% difference in the three kinds of specimens, suggesting that the effect of β-component is not large enough to change our conclusion. More accurate results would be obtained if γ-component was included although it would have minor effects on SHG.

Reportedly, the ratio of type III collagen in the tendon was less than 5%^35^, which is smaller than our result (13.1% ± 3.8%). One possible cause for this might be the way to select the area of type III band in SDS-PAGE gel. Because the band of the rat tail-tendon (lane 4 in Fig. 4) was not clearly recognized, we defined the type III region in reference to the purified type III band (lane 3 in Fig. 4). In this process, we might have overestimated the type III band region. Other possible factor is the background noise of SDS-PAGE staining. As described in the method section, the intensity of the whole image was subtracted by the background value. It however remains unclear if the background noise of SDS-PAGE staining is totally canceled.

For SDS-PAGE analysis, samples were treated with pepsin, the solubilized collagen was precipitated and subjected to SDS-PAGE analysis. The data of the *λ*_*III*_ obtained with the SDS-PAGE was compared with the *λ*_*III*_ obtained by the proposed method that uses SHG light intensity, and they were quantitatively in good agreement. These *λ*_*III*_ were also congruent with Mccullagh and Balian^21^ that showed the ratio of type III and I to total collagen in the human aortic media are 70% and 30%, respectively. Only soluble collagens were however analyzed in Mccullagh and Balian^21^. According to Menashi et al^36^, type I/III collagen ratio is 2:1 if the whole collagen including insoluble ones is analyzed. Since *λ*_*III*_ obtained with the proposed method is close to the type I/III ratio of soluble collagens, the proposed method may quantify the type I/III ratio of soluble collagens.

At the beginning of this study, we attempted to quantify *λ*_*III*_ from the difference in SHG light intensity recorded on the photon multiplier tubes between the backward and forward directions, since the forward–backward ratio of SHG light intensity was reported to decrease with increasing type III collagen concentration^19^. However, the forward–backward ratio was almost constant regardless of *λ*_*III*_ gels (Fig. 2n). Since the strength of SHG intensity is affected by the width of fiber in the forward direction, thinner fibers in the gels might have led to the constant value of the forward–backward ratio. Therefore, when using SHG light intensity to measure *λ*_*III*_, the backward direction is more suitable.

In conclusion, we propose a novel method to quantify the ratio of type III to total collagen in various tissues, based on SHG light intensity and the diameter of collagen fiber bundles. We applied this method to collagen fiber bundles in the media and adventitia of porcine thoracic aortas and confirmed its measurement accuracy and suitability by comparing data obtained with SDS–PAGE.

## Methods

### Quantification of the relationship between SHG light intensity and ***λ***_***III***_

Type III collagen solutions of various *λ*_*III*_ were prepared by mixing 3 mg/mL of type I (PSC-1-100-20, Nippi, Tokyo, Japan) and type III (PSC-3-100-20, Nippi) collagen solutions in ratios of 5:0, 4:1, 3:2, 2:3, 1:4, and 0:5. A silicone rubber container (internal dimensions: 15 × 24 × 3 mm) was placed on a glass slide and cleaned with ethanol. Then, 1–5 µL of 1 M NaOH filtered with a φ0.20-µm filter was added to each collagen solution to obtain a final concentration of 0.1%, and 1 mL of each mixed solution was poured into the container and incubated at 37 °C for 24 h to obtain a gel. Thereafter, the container was removed and a coverslip was placed on the collagen gel to prepare the observation sample. A control sample was prepared using 1 mL of incubated phosphate-buffered saline [PBS(−)].

SHG light intensity of the collagen was imaged under a multiphoton microscope (FV1200MPE, Olympus, Tokyo, Japan), as reported previously^18, 37^. Briefly, a laser (wavelength, 800 nm; pulse width, 100 fs; repeated frequency, 80 MHz) was applied to the prepared gels through a 25 × objective lens (NA = 1.05, XLPLN25XW, Olympus). The generated SHG light was observed using a bandpass filter (400 ± 5 nm). Data were recorded on photomultiplier tubes in both forward (transmitted) and backward (reflected) directions. Observation conditions were the same for all specimens (HV 600 V, laser power = 18%, dwell time = 10 µs/pixel, Karman filter = 2). To reduce the influence of structural variations of collagen fiber such as direction and dimeter, we imaged 51 slices with an interval of 2.0 µm at 5 positions per specimen. Five specimens were tested for each experimental condition, and the average was used.

Image analyses were performed using ImageJ (v. 1.51i, National Institute of Health, Bethesda, MD, USA). From the image stack, 40 slices, which cover the whole thickness, were selected and the average intensity in the image stack *I*_*gel*_ was measured. *I*_*SHG-λIII*_ was determined by subtracting the background intensity in the SHG image *I*_*BG*_, as follows:

10 $$I_{SHG - \lambda III} = I_{gel} {-}I_{BG} .$$

Finally, fitting constants *k*_1_ and *k*_2_ in Eq. (2) were determined using the least-squares regression from the relationship between *I*_*SHG-λIII*_ and *λ*_*III*_.

### Quantification of the relationship between SHG light intensity and the diameter of type I collagen fiber bundles

All animal experiments were approved by the Institutional Review Board for Animal Care of the Nagoya Institute of Technology, following recommendations from their Guide for Animal Experimentation and this study is reported under ARRIVE guidelines (https://arriveguidelines.org). Three male Wistar/ST rats (15-week-old, Chubu Kagaku Shizai, Nagoya, Japan) were used in this experiment. After the rats were euthanized using CO_2_, their tail tendons were excised and collagen fiber bundles (*D* = 5–100 µm) were extracted using forceps. Both ends of the fiber bundle were glued on a polyethylene terephthalate (PET) film sheet with a cyanoacrylate adhesive. The samples were then sent to a laboratory-made tensile tester^38^ to stretch the specimen. When the tester recorded 2 mN of force, the specimen was deemed to have been sufficiently stretched. We selected a small sample size to reduce the animal experiments but multiple animals to consider the individual difference (three rats).

SHG light intensity from a collagen fiber bundle was imaged under a multiphoton microscope (FV1200MPE, Olympus) using a 5 × objective lens (MPLN5X, NA = 0.1, Olympus). In this condition, image depth was 109.3 µm. Thus, a single slice was considered to include all depth-wise SHG light generated from a collagen fiber bundle with a diameter several 10 µm. However, because the long axis of the collagen fiber bundle might not be parallel to the image plane, several slices were imaged with 10-µm intervals. Observation conditions were the same for all specimens (HV, 250 V; laser power = 18%; dwell time = 10 µs/pixel; Karman filter = 2). After measuring the SHG light intensity, the fiber bundle was imaged again using a 5 × optical zoom function in addition to the 5 × objective lens to measure its exact diameter.

The maximum intensity projection image was obtained from the stacked image of the fiber bundle. The fiber bundle region was defined and the average SHG light intensity in the region *I*_*F*_ was recorded from triplicate measurements. Similarly, average SHG light intensity in the background area *I*_*BG*_ was measured in triplicates, and the SHG light intensity generated from the tendon collagen fibers *I*_*SHG-Tendon*_ was determined as follows:

11 $$I_{SHG - Tendon} = I_{F} {-}I_{BG} .$$

From the fiber image obtained using the 5 × optical zoom function, the diameter was manually measured at any three locations and its average was determined as fiber diameter *D*. From the relationship between *I*_*SHG-Tendon*_ and *D*, constant *k*_3_′ was determined in Eq. (7) using a least-squares regression analysis.

### Measurement of ***λ***_***III***_ in a collagen fiber bundle obtained from porcine thoracic aorta using SHG light intensity

Porcine thoracic aortas were purchased from a local slaughterhouse (Handa meat market, Handa, Japan) and transported to our laboratory in PBS( −) at 4 °C. The connective tissues were removed with forceps, and the ventral side of the aorta between the first and the second intercostal arteries from the proximal side was dissected using a surgical knife. The adventitia was separated from the aorta with forceps, while the medial specimens were cut into blocks (5 mm in the longitudinal and 10 mm in the circumferential directions), immersed in a 6% w/w agar solution, and kept at 4 °C for 30 min. The specimens were then sliced into 100-µm sections using a micro slicer (DTK-1000, Dosaka-EM, Kyoto, Japan). Adventitial and medial samples were separated into in microtubes and immersed in 68.5 units/mL of elastase (120–145 units/mg, ES438, EPC, Owensville, MO, USA) in Hanks balanced buffer (pH 7.1–7.5, HBSS(−)) for 2 h at 37 °C under shaking at 60 rpm to purify the collagen fiber bundles. Finally, the samples were washed with PBS(−) thrice.

From the obtained specimens, collagen fiber bundles were excised with forceps. Similarly to that stated above, each bundle was glued on a PET film and stretched and SHG light intensity (*I*_*SHG*_) and diameter *D* were measured. Finally, *λ*_*III*_ was calculated from Eq. (6).

### SDS–PAGE

The molecular weight of type I collagen (283.3 ± 1.3 kDa) is similar to that of type III (288.3 ± 1.3 kDa)^20^. Therefore, we added urea in the gel to distinguish the band positions of type I and III collagens, as described previously^39^.

Collagen fiber bundles from rat tail tendons and porcine aortic media and adventitia samples were obtained, as described above, and specimen solutions were obtained as described previously^40^. Specimens were homogenized in 20% w/v NaCl in 0.05 M Tris buffer (pH 7.5) using an ultrasonic homogenizer (VP-050 N, TAITEC, Koshigaya, Japan). The samples were centrifuged and the sedimentation was resuspended in 0.6 mg/mL pepsin (P7012, Sigma-Aldrich, St. Louis, MO, USA) and 0.5 M acidic solution for 24 h. The solution was centrifuged and its supernatant was dialyzed overnight in 0.02 M disodium hydrogen phosphate (pH 9) using a 20-kDa cassette (87,735, Thermo Fisher Scientific, Waltham, MA, USA). The dialyzed solution was centrifuged, and the sediment was obtained as a sample for SDS–PAGE.

The sample buffer used for SDS–PAGE comprised 62.5 mM Tris buffer (pH 6.8), 2% SDS, 25% glycerol, and 0.5% bromophenol blue. The prepared collagen samples were dissolved in an equal volume of sample buffer and 0.5 M Tris buffer (pH 6.8) to obtain a final concentration of 50 mg/mL. Additionally, 3 mg/mL of type I (PSC-1-100-20, Nippi, Tokyo, Japan) and type III collagen (PSC-3-100-20, Nippi, Tokyo, Japan) solutions were separately prepared for use as control samples for type I and III collagen. After adding 2-mercaptoethanol to obtain a 5% concentration, the solution was spun down, incubated at 100 °C for 5 min, and centrifuged.

A separation gel was prepared using 7.5% acrylamide /N,Nʹ-methylenebis(acrylamide), 0.37 M Tris buffer (pH 8.8), 0.1% SDS, 2 M urea, 0.05% tetramethylethylenediamine, and 0.1% ammonium persulfate. A stacking gel was also prepared using 4% acrylamide/N,Nʹ-methylenebis(acrylamide), 0.06 M Tris buffer (pH 6.8), 0.1% SDS, 2 M urea, 0.05% tetramethylethylenediamine, 0.1% ammonium persulfate. The composition of the running buffer was 25 mM Tris base, 19 mM glycine, and 0.1% SDS.

The sample was electrophoresed at 200 V for 100 min and proteins were visualized with Coomassie Brilliant Blue R-250. The gel was imaged using ChemiDoc MP Imaging System (Universal Hood III, Bio-Rad). SDS–PAGE was performed in triplicates using different samples.

For image analysis, the intensity of the whole image was subtracted by the background value. Then, band regions were selected in reference to type I (lane 2 in Fig. 4) and type III samples (lane 3 in Fig. 4) and the intensity of each region was summed. Although dimers (β-component) and trimers (γ-component) have been reported previously^41^, regions of α_1_(I)- and α_2_(I)-chains and β(I)-components were considered type I collagen and those of α_1_(III)-chain and β(III)-component were considered type III collagen. The measurements were performed in triplicates for each gel.

### Statistics

Pearson’s correlation coefficient was calculated for the plots between the *I*_*SHG-λIII*_–*λ*_*III*_, forward/backward ratio–*λ*_*III*_, *I*_*SHG-Tendon*_–*D*, and *I*_*SHG-TypeI*_–*D*. *P* values of < 0.05 were considered statistically significant. Data were averaged and presented as mean ± standard deviation (SD). All analyses were performed using Microsoft Excel (2016).

## Supplementary Information

Supplementary Information.

## Data Availability

The datasets generated during and/or analyzed during the current study are available from the corresponding author on reasonable request.
