# Supplementary material for: Second harmonic generation light quantifies the ratio of type III to total (I + III) collagen in a bundle of collagen fiber
Source: Sci Rep. 2021 Jun 4;11:11874. doi: 10.1038/s41598-021-91302-3 (PMC8178339; doi:10.1038/s41598-021-91302-3)
Supplement: Supplementary file 1 — Supplementary Information. [file 41598_2021_91302_MOESM1_ESM.docx]

**Second harmonic generation light quantifies the ratio of type III to total (I + III) collagen in a bundle of collagen fiber**

Shukei Sugita ^1, 2)^*, Takuya Suzumura ^1)^, Akinobu Nakamura ^3)^, Shinya Tsukiji ^3, 4)^, Yoshihiro Ujihara ^1)^, and Masanori Nakamura ^1, 2, 4)^, *Nagoya, Japan*

**Affiliation:**

1. Department of Electrical and Mechanical Engineering, Graduate School of Engineering, Nagoya Institute of Technology
2. Center of Biomedical Physics and Information Technology, Nagoya Institute of Technology
3. Department of Life Science and Applied Chemistry, Nagoya Institute of Technology
4. Department of Nanopharmaceutical Sciences, Nagoya Institute of Technology

***To whom correspondence should be addressed:**

Shukei Sugita, Ph.D.

Department of Mechanical Engineering, Graduate School of Engineering, Nagoya Institute of Technology, Gokiso-cho, Showa-ku, Nagoya 466-8555, JAPAN

Tel. and Fax: +81 52 735 7125

E-mail: [sugita.shukei@nitech.ac.jp](mailto:sugita.shukei@nitech.ac.jp)


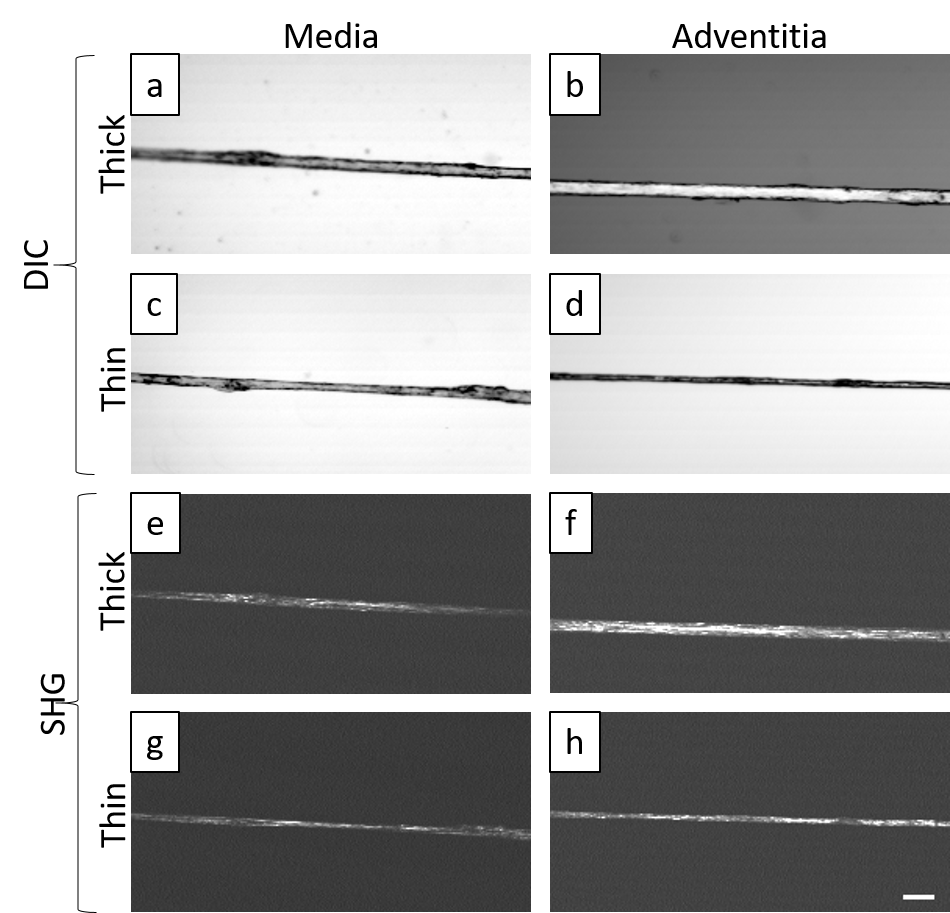


**Fig. S1** Typical images of medial and adventitial collagen fiber bundles. (a–d) Differential interference contrast (DIC) images and (e–h) SHG images of (a, b, e, f) thick and (c, d, g, h) thin fiber bundles. Samples were obtained from (a, c, e, g) media and (b, d, f, h) adventitia of porcine thoracic aorta. Scale bar = 100 μm.


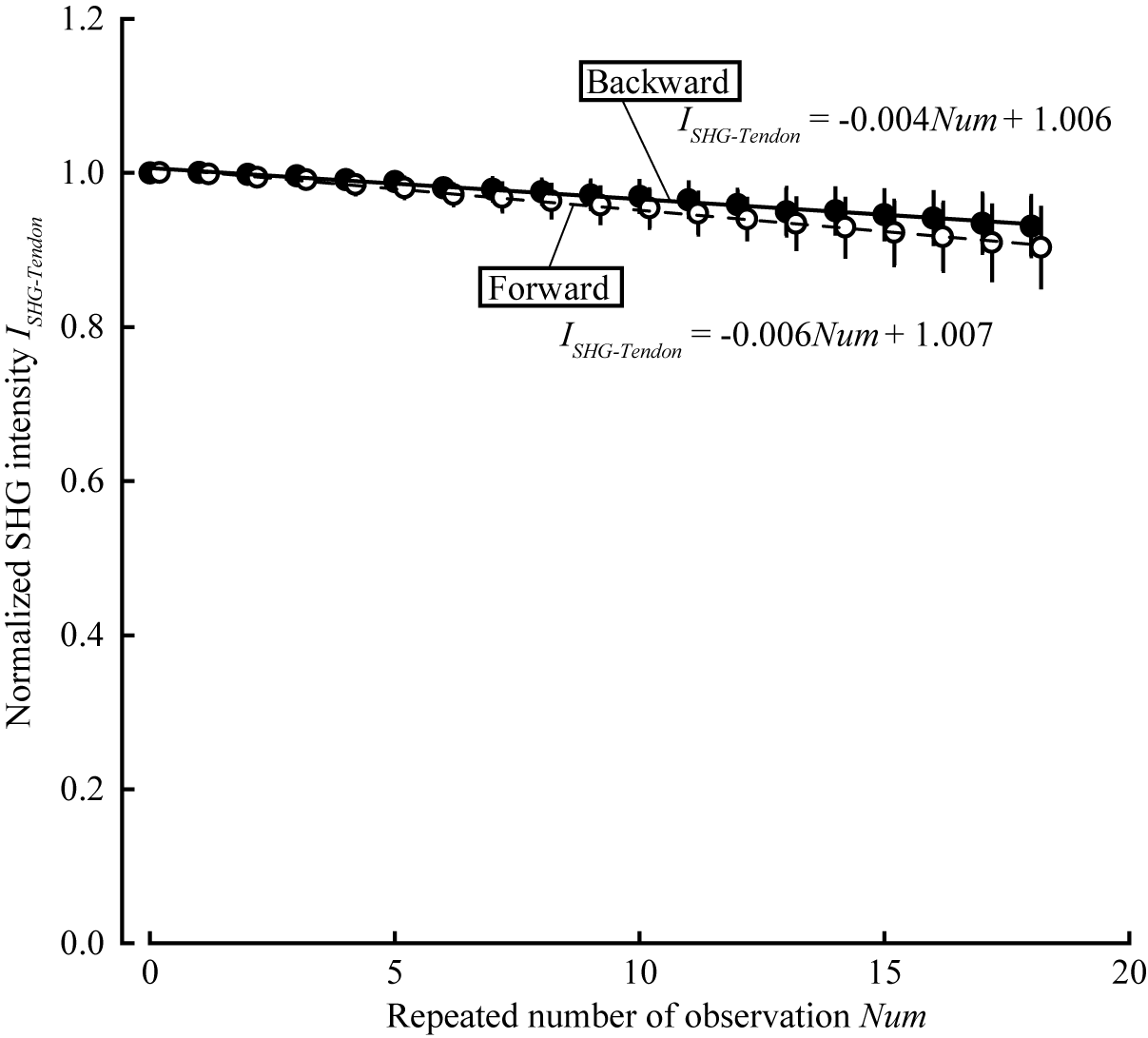


**Fig. S2** The decrease in SHG intensity of collagen fiber bundles after repeated measurements. Loss in SHG intensity after repeat measurements of the same fiber bundle. Data are shown as mean ± SD. *n* = 3.
